# Assessing the cognition, attitudes and intentions of volunteers regarding unrelated peripheral blood stem cell donation: The UPBSC-DQ instrument in Chinese

**DOI:** 10.1016/j.heliyon.2023.e20663

**Published:** 2023-10-20

**Authors:** NaNi Ding, ZhuoNi Ye, XinQian Jin, GuoHua Zhang, QiuLin Yu, YuPeng Liu

**Affiliations:** a Wenzhou Vocational College of Science and Technology, Wenzhou, Zhejiang, China; b The Second Affiliated College of Clinical Medical, Wenzhou Medical University, Wenzhou, Zhejiang, China; c College of Psychiatry, Wenzhou Medical University, Wenzhou, Zhejiang, China; d The First Affiliated College of Clinical Medical, Wenzhou Medical University, Wenzhou, Zhejiang, China; e Department of Epidemiology and Biostatistics, School of Public Health and Management, Wenzhou Medical University, Wenzhou, Zhejiang, China

**Keywords:** Donation, Unrelated peripheral blood stem cell transplantation, Questionnaire, Reliability, Validity

## Abstract

**Objectives:**

This study aimed to develop and validate the Unrelated Peripheral Blood Stem Cell Donation Questionnaire (UPBSC-DQ) (an instrument in Chinese) to assess the degree of cognition, attitude and intention of enrolled volunteers towards UPBSC donation.

**Methods:**

The development process of the UPBSC-DQ was performed in a stepwise approach that included extensive literature retrieval, expert revision, and pretesting with 442 students. We conducted an online cross-sectional survey using the final version of the UPBSC-DQ among 336 participants. The reliability of the questionnaire was assessed by Cronbach's α and corrected item-total correlation (CITC), and the validity was evaluated by a correlation coefficient matrix, confirmatory factor analysis (CFA), and *t*-test.

**Results:**

The UPBSC-DQ consists of four domains: basic information, cognitive, attitude, and intention. The Cronbach's α values were 0.88 and 0.86 for the attitude and intention scales, respectively, indicating strong internal consistency and good reliability. Correlation analysis and CFA showed good structure and content validity. Interitem correlations indicated that each item had only a weak correlation with the other scales.

**Conclusions:**

The UPBSC-DQ is a reliable and valid assessment questionnaire for individuals’ attitudes and intentions towards UPBSC donation. The questionnaire showed good to high reliability, content and construct validity.

## 1 Introduction

Haematopoietic stem cell transplantation (HSCT) is a curative procedure for many life-threatening oncologic, haematologic and immunologic disorders [1]. Over the past decade, peripheral blood stem cells (PBSCs) have replaced bone marrow (BM) as the main source of HSCs [2] due to easier collection, better recovery capability, and improvements in overall survival [3,4]. Ideally, patients receive PBSCs donated by a matched blood relation (e.g., a sibling). However, as there is only a 25 % chance for a sibling to be a matched human leukocyte antigen (HLA), only one-third of patients have a matched sibling donor [1]. For other patients, the only available option is an unrelated HLA-matched donor that can be identified through volunteers [5]. Michael et al.’s study (2019) showed that related PBSC donors are at increased risk for donation-related toxicities compared with unrelated donors one year after donation [6]. However, the likelihood of finding such donors is low because of insufficient or nonexistent national donor programmes in many developing countries [7].

Therefore, the HSCT waiting list is expanding around the world because the need is greater than the number of registered volunteers [8]. The gap between need and donation is especially wide for unrelated HSCT in many developing countries, such as China. UPBSC donors have been the main composition of BM banks with a large expansion space. However, due to limited cognition of UPBSC donation (UPBSCD), people often assume that it is the same or carries the same risk as BM donation, and few people are willing to accept recruitment. Furthermore, the rate of attrition for UPBSCD registers at the marrow bank is very high due to the selectivity of enrolment and the complete autonomy of the decision to donate [9]. In other words, the rate of regret prior to donation is high.

To achieve donor retention, it is essential to identify factors that positively or negatively affect the decision to donate. Ding et al. (2018) examined related factors for intention towards PBSC donation via a set of questionnaires and found that more knowledge and a more positive attitude towards UPBSCD were significantly and positively associated with stronger donation intention [10]. To the best of our knowledge, this was the first study to show that enhancing knowledge, intention and attitude towards UPBSCD may be helpful to promote the possibility of donation and reduce the high rate of registry attrition related to UPBSCD. The study demonstrated that more complete cognition was conducive to eliminating negative emotions, forming a positive donation attitude, and ultimately affecting the intention and motivation to donate. A study demonstrated the effectiveness of the protocol guiding stem cell donor recruitment at drives to recruit the most-needed donor demographics and quality donors [11]. However, neither individual behaviour nor the prevalence of the motivation to donate are homogeneous [12].

There is therefore an urgent need for an instrument to assess the cognition, attitude, and intention of registered volunteers for UPBSCD. Questionnaires are the most commonly used tool because they are reliable, relatively inexpensive, and easy to use in a short time for a large number of people. Previous studies have assessed related factors (e.g., donation of blood products, medical history of blood cancer) of HSC donation among volunteers [13]. It is urgent to explore deeper influencing factors related to individuals, families, health care systems and governments to identify targets for promoting UPBSCD. Few scales have been designed to assess the cognition, attitude, and intention towards UPBSCD among registered volunteers in developing countries.

In this study, we developed the Unrelated Peripheral Blood Stem Cell Donation Questionnaire (UPBSC-DQ) and validated the reliability and validity of this questionnaire to assess the cognition, attitude and intention towards UPBSCD. This study investigated and analysed the cognition, attitude and intention towards UPBSCD and related populations and did not consider stem cell transplantation from related donors, BM or cord blood.

## 2 Materials and methods

### 2.1 Participants

The study population was composed of 336 college students and teachers at Wenzhou University Town (WUT) in Wenzhou, China. Participation in the study was voluntary, and careful consideration was used in reporting the findings to protect student confidentiality. Written consent was obtained from all participants. Ethical approval of the present study was obtained from The Red Cross Society in Wenzhou.

A cross-sectional survey was created using WenJuanXing Online Survey Software (https://www.wjx.cn/vj/O8pPPJA.aspx). In this study, we primarily focused on attitudes and intentions towards donation; therefore, the total score of donation attitudes and intentions was the main outcome. In the pretesting of this questionnaire, σ (the standard deviation) of the total score for attitudes and intention was 8.0 and 2.5, respectively. When we used attitudes as the main outcome in the calculation of the sample size, δ (the allowable error) was set as 1.5, α was set as 0.05, and $u_{\alpha }$/2 was 1.96. We used Formula n = ${\left(\frac{u_{\alpha /2}S}{\delta }\right)}^{2}$ (where S is the standard deviation (SD) of the total score of the sample) and found that n was 109. Due to the convenience sampling used in this study, we set the design effect *Deff* parameter as 3.0 (*Deff* = 3.0); thus, the total sample size required in this study (N = n × *Deff*) was 327. When using intention as the main outcome, we found that n was 96 and N was 288. We selected the larger of the sample size values obtained from the above two calculations. Therefore, the final sample size was 327 in this study.

To ensure the structure and assessment of the questionnaire, the second version was tested online from November 28, 2020, to December 2, 2020. We posted three promotional posters on the university campus, invited students and teachers to participate in the survey voluntarily, and collected a total of 384 complete questionnaires through the online platform of the Wenjuanxing survey. After quality control, 48 questionnaires were excluded, leaving 336 valid questionnaires for analysis with a response rate of 87.50 % (excluding questionnaires with a response time of less than 180 s because we believed this was too short and could result in a large deviation from the real situation that would be beyond the allowable range and would have the potential to influence the final outcome). The effective questionnaire analysis results of the pretest showed that the average response time of the questionnaire was 416.1 ± 236.7 s (median value: 346.0, range: 264.0 to 482.3), and the 95 % confidence interval (CI) was 390.7–441.5 s.

### 2.2 Procedures

We first obtained permission from the Ethics Committee of Wenzhou Medical University (Wenzhou, China) to recruit individuals willing to participate in this study. The study was conducted following the Declaration of Helsinki and all applicable local and international ethical guidelines. Participants were informed about the study's purpose, procedures, risks, and benefits. We ensured them that their participation was voluntary and that they could withdraw from the study at any time without any consequences. The ethics code for this study is 2019-109 (**Supplementary File 3**).

### 2.3 Instrument development

**Fig. 1:**
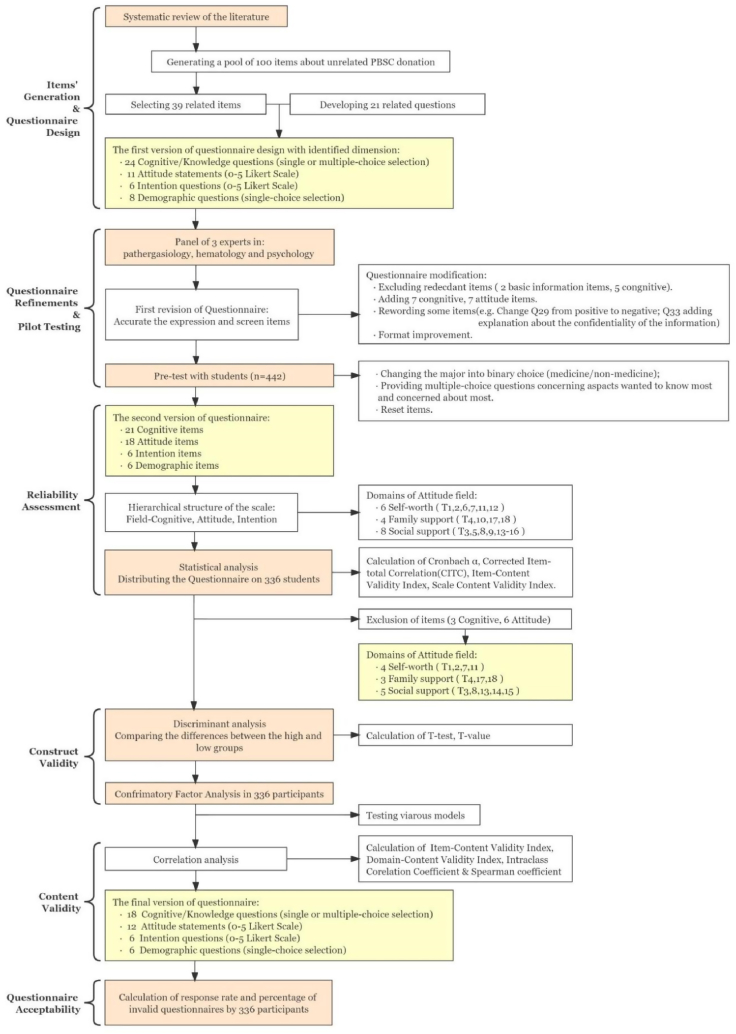
Flow diagram describing the steps followed to develop and validate the UPBSC-DQ. Abbreviations: PBSC, peripheral blood stem cell; UPBSC-DQ, unrelated peripheral blood stem cell donation questionnaire.

The instrument was developed in four standard steps: (i) item generation and questionnaire design; (ii) initial item refinements and pilot testing; (iii) identification of domains, reliability and validity assessment of scales; and (iv) scale finalization and acceptability calculation. Fig. 1 shows the process of the questionnaire design and verification in detail.

#### 2.3.1 Phase 1 | Item generation and questionnaire design

First, a research team was set up that consisted of a panel of experts, college instructors and Red Cross staff as professional advisors. Students and teachers in the WUT were respondents. The expert panel comprised three people: Professor DeHui Lau (Fellow of the Royal College of Physicians, The Chinese University of Hong Kong, specializing in psychiatry), Professor Kang Yu (The First Affiliated Hospital of Wenzhou Medical University, who performed the first successful HSCT in southeast Zhejiang, specializing in haematology), and Professor Xin Yu (Director of the Institute of Mental Health, Peking University, specializing in psychology).

After systematically reviewing the relevant literature, the researchers generated a pool of 100 optional items about UPBSCD based on 50 questions on the database of UPBSC donors in China and other questionnaires, such as the questionnaire for retrospective analysis after PBSCD [14], the Mayo-designed MS questionnaire [15], the questionnaire of Public Awareness of Cord Blood Banking [16], KAP modelled questionnaires [17], and CTXD [18]. After removing duplicates or similar questions, we screened 39 related items with minor modifications to make them more suitable and precise for our participants. In addition, we developed 21 extra questions about UPBSCD, which mutually constituted the contents of the initial questionnaire divided into cognitive, attitude, intention and demographic sections. Cognition and attitude tend to be highly correlated [19]. The questionnaire evaluates the following three dimensions: cognition/knowledge (what the participants know about UPBSCD and leukaemia), attitude (what the participants consider or believe about UPBSC transplantation and leukaemia) and intention (what they do regarding UPBSC transplantation and leukaemia).

#### 2.3.2 Phase 2 | initial item refinements and pilot testing

The dimensions and individual items were assessed by the panel of 3 experts mentioned above. After several rounds of modification, we excluded 7 redundant items and added 7 cognitive items. Seven attitude items were reworded, and several items were formatted (Fig. 1).

Accordingly, we conducted a pretest on 442 college students using the first version of the questionnaire in phase 1 to assess its readability and comprehensibility, whether it achieved expectations, and its relevance to our purpose. Based on the results of these interviews, we refined several items in the questionnaire. The second version of the questionnaire included 6 demographic, 21 cognitive, 18 attitude and 6 intention items (**Supplementary File 1**).

#### 2.3.3 Phase 3 | identification of domains, reliability and validity assessment of scales

The final version of the questionnaire was distributed to 336 participants in the WUT. We converted the dimensions of cognition, attitude and intention into three scales (Fig. 1 and Supplementary File 1A). Emphasis was placed on the attitude and intention scales. The domains covered attitudes originating from self-worth (including 6 items), family support (4 items) and social support (8 items).

The internal consistency reliability of this scale was assessed using the Cronbach's alpha coefficient. Based on the 336 respondents, we calculated alpha values for all scales. Construct validity assesses the extent to which a questionnaire measures the construct of an object. A *t*-test and confirmatory factor analysis (CFA) were used to assess the construct validity. Donation intention was measured via 6 items assessed on a 3-point scale. Cronbach's α was 0.86. The construct validity was good (P < 0.001, GFI = 0.92).

The items of the attitude scale were selected via Cronbach's α and CITC. An alpha range of 0.7–0.9 was considered acceptable because a higher score can indicate item redundancy [20,21]. The Cronbach's α coefficient of the total initial 18 items was 0.82. After deleting each item and cultivating the remaining α, we considered the item removable if the remaining α was >0.82. The second standard was that items whose CITC was less than 0.3 were considered removable. When ranking items that meet the two criteria simultaneously, the priority for deletion was given to the item with the largest increase in α and the smallest relative CITC. We repeated the above steps until neither criterion was met. Ultimately, we removed 6 items (T5, T6, T9, T10, T12, T16) and retained 12 standardized items to construct the attitude scale. The final version of the questionnaire is provided in **Supplementary File 2**, submitted in the form of a web version as a self-administered questionnaire (https://www.wjx.cn/vj/O8pPPJA.aspx).

#### 2.3.4 Phase 4 | scale finalization and acceptability calculation

We report results from the quantitative test of reliability and validity for selected domains and dimensions. We calculated the response rate and percentage of invalid questionnaires for 336 participants. Based on this, the final version of the cognitive, attitude and intention measurement scale for UPBSCD was ultimately formed with high feasibility and good reliability and validity.

### 2.4 Questionnaire composition

The final questionnaire consisted of four sections: demographics, cognition, attitude, and intention. The demographic information included age, gender, and major. The cognitive section included 18 items, each of which had two “yes or no" options. A correct answer earned 1 point, and an incorrect answer earned 0 points. The total score was between 0 and 18 points. The attitude section included 12 items, with each item scored on a 5-point Likert scale (1 = strongly disagree, 5 = strongly agree). The total score of the attitude scale ranged from 12 to 60, with a higher score indicating a higher level of attitude. The intention section included 6 items, with each item scored on a 3-point Likert scale (1 = I will not, 2 = I am not sure. , 3 = I am willing). The total score of the intention scale ranged from 6 to 18, with a higher score indicating a higher level of intention. The research team then examined the validity and reliability of the selected dimensions to test the final version of the questionnaire (**Supplementary File 1**). Data were gathered on December 2, 2020.

### 2.5 Statistical analysis

After collecting questionnaires on the WenJuanXing website (https://www.wjx.cn/vj/O8pPPJA.aspx), the data were first imported into Microsoft® Excel® 2019MSO (Microsoft, NY, USA). Statistics were performed using IBM SPSS Statistics version 25.0 (IBM, Corp., Armonk, NY, USA), IBM SPSS AMOS 21.0 (IBM Corp., Armonk, NY, USA) and GraphPad Prism® version 9.0.0 for Windows (GraphPad, La Jolla, CA, USA). The statistical significance for all analyses was set at 0.05.

In this study, we performed normality tests on all numeric variables. If the data were normally distributed, we used the mean (± standard deviation, SD) for analysis, and if not, we used the median (interquartile range, IQR). For categorical variables, we used the number (percentage) for analysis.

When analysing group differences, we tested the assumption of variance homogeneity for numeric variables that met the normality assumption. If the variance was homogeneous, we used a *t*-test to analyse the data; if not, we used a corrected *t*-test. For numeric variables that did not meet the normality assumption, we used nonparametric tests ((Mann‒Whitney *U* test). For categorical variables, we used the chi-square test to analyse the data.

#### 2.5.1 Descriptive statistical analysis

First, we conducted a descriptive statistical analysis of the cognitive dimension for age and gender by computing the percentages, means, and standard deviations of demographic information to describe the characteristics of the participants with 95 % confidence intervals (CIs) when applicable. Emphasis was placed on assessing scales of attitude and intention dimensions.

#### 2.5.2 Reliability

The internal consistency reliability of the scales (attitude and intention) was assessed using Cronbach's α coefficient. A threshold value of 0.70 was expected [22]. The α statistic tends to underestimate reliability if items are inefficient or do not measure the same index and content in a scale (not homogenous). We calculated α values for the selected scales and deemed values > 0.70 to be a satisfactory level of internal consistency.

We had previously divided the attitude field into three domains (self-worth, family support and social support). We screened the attitude items via 2 standards, CITC and Cronbach's α coefficient values of other items except individual items. CITC can be used to assess internal consistency and reliability analysis. The higher it is in a test result, the higher the α value [23]. CITC <0.3 is considered unacceptable. If the α value, except for certain items, is lower than the included one, it is acceptable to delete the item. Items that met both criteria were removed one by one, and 12 items were screened to conduct domain-reliability analysis. Cronbach's α based on standardized items >0.7 indicates that the reliability of the scale is good to excellent [24]. The percentage of respondents who chose the highest and lowest scores on the scale was less than 25 %, indicating a parametric distribution and a normally distributed bell-shaped curve, which suggests that the data are reliable.

#### 2.5.3 Construct validity

**2.5.3.1 Discriminant validity.** We calculated the *t*-test as a primary method to assess the main differences in scores in multiple domains of the same nature. A *t*-test was used to compare the difference between the highest subgroup (in descending order of scores, it was in the top 33.3 %) and the lowest subgroup (in the bottom 33.3 %) to investigate the discrimination ability of the scales. In accordance with the central limit theorem, a dataset of 336 participants that met the requirements of normality was necessary for the normality assumption. The presence of ceiling/floor effects was defined as 25 % or more of respondents having the highest or the lowest possible score on the attitude and intention scales, respectively [25,26], the higher proportion of which indicated that the instrument was unable to distinguish the concept.

**2.5.3.2. Factorial validity.** Factorial validity is an empirical assessment of construct validity using factor analysis statistical models. A factor is a combination of items that measure the same dimensions. This step was conducted when the questionnaire was constructed to measure more than one dimension. The Attitude and Intention scale was designed to comprise two dimensions of 18 items (Fig. S1). The attitude dimension was divided into three domains (self-worth, social support, and family support), and the intention dimension was divided into two domains (self-worth and family support). CFA was conducted on separate subsamples and assessed the relationships between items and their corresponding factor. The normality of the distribution of each item included in the two dimensions was explored, and CFA was performed using the maximum likelihood robust estimation method. We assessed the goodness of fit of the models using the following statistics: χ2/df (degrees of freedom), Comparative Fit Index (CFI), Bentler-Bonett Normed Fit Index (NFI), Incremental Fit Index (IFI), Tucker‒Lewis Index (TLI), Root Mean Square Residual (RMR), Goodness-of-Fit Index (GFI), Root Mean Square Error of Approximation (RMSEA) [27], Akaike information criterion (AIC), Browne-Cudeck criterion (BCC), Bayesian information criterion (BIC) and Consistent Akaike Information Criterion (CAIC) [28]. AIC, BCC, BIC and CAIC were used as model fit criteria and to select the best hypothesis from a set of competing theory-based hypotheses [29]. CFI ≥0.9, NFI ≥0.9, IFI ≥0.9, TLI ≥0.9, RMR <0.09, GFI ≥0.9, and RMSEA <0.100 were considered acceptable fit, and CFI ≥0.95, RMR <0.05, GFI ≥0.9, RMSEA <0.080 and closer to 0.050 were considered satisfactory fit [27].

#### 2.5.4 Content validity

Content validity was examined by comparing item-item or item-domain data with the nonparametric Spearman correlation coefficient and its 95 % CI. A correlation coefficient matrix was used to assess interitem correlation and item-scale correlation by making pairwise comparisons of items or domains [30]. According to Dominguez-Olivan, the standard of correlation (r value) distribution is as follows: little or no relationship (r = 0–0.25), fair relationship (r = 0.25–0.5), moderate relationship (r = 0.5–0.75), and very good to excellent relationship (r > 0.75) [31].

## 3 Results

### 3.1 General characteristics of the study subjects and level of variables

The characteristics of 336 participants are shown in Table S1. Of the 336 effective subjects who participated in the study, the majority (69.3 %, 233/336) were female. The mean age was 19.15 years (SD 8.74 years), and most subjects were 17–20 years old (98.2 %, 330/336). Nearly half (42.6 %, 143/336) were educated in medicine and paramedicine, with 57.4 % (193/336) majoring in other fields. The mean score of the cognitive scale was 11.18 (SD = 1.94; 95 % CI = 10.98 to 11.39).

### 3.2 Reliability

Table 1 shows the internal consistency reliability results of the attitude and intention scales. The total scores of the attitude and intention scales were 66.96 (SD 8.32) and 9.19 (SD 2.60), and Cronbach's α for attitude and intention was 0.88 and 0.86, respectively. The items of the attitude scales were divided into 3 domains: self-worth, social support and family support (**Supplementary File 1B&C**), for which the mean scores were 14.49 (2.78), 20.13 (2.98), and 11.98 (2.07), respectively, showing that the difference in means apparently differed across the three domains. The Cronbach's α values for each attitude domain were all larger than 0.7, which showed acceptable reliability for domain classification (Table S2). Moreover, the Cronbach's α of the remaining items on the corresponding scale was excellent (>0.85), showing excellent internal consistency of the scales. The intention questions also showed excellent internal consistency (>0.80) (Table 1). Additionally, among all the subjects, 6 (1.7 %) and 1 (0.2 %) received the highest score and the lowest score, respectively, and neither exceeded 25 %, so there was no ceiling/floor effect (Table 1).

**Table 1:** **-** Reliability assessment of attitude and intention scales.

Domain	No. of Items included	Mean (SD)	Cronbach's α	Cronbach's α Based on Standardized Items	Minimal (%)	Maximal (%)
Attitude						
Total	12	46.60 (6.71)	0.88	0.88	1 (0.3)	9 (2.7)
Self	4 (T1, T2, T5, T7)	14.49 (2.78)	0.75	0.75	1 (0.3)	22 (6.5)
Social	5 (T3, T6, T8, T9, T10)	20.13 (2.98)	0.71	0.72	29 (8.6)	30 (8.9)
Family	3 (T4, T11, T12)	11.98 (2.07)	0.72	0.72	2 (0.6)	50 (14.9)
**Intention**						
Total	6	14.81 (2.60)	0.856	0.854	1 (0.3)	74 (22.0)

Abbreviations: SD, standard deviation.

### 3.3 Construct validity

#### 3.3.1 Discriminant validity

Table 2 shows the results for discriminant validity. For attitude, the total score of the lowest subgroup was significantly lower than that of the highest subgroup (38.99 vs. 54.59, *P* < 0.001), suggesting a significant degree of discriminant validity. For all three domains of attitude, similar results were obtained. For intention, the median value of the lowest subgroup was significantly lower than that of the highest subgroup (12 vs. 18, *P* < 0.001), also indicating that the discriminant validity of the scales was very good.

**Table 2:** **-** Discriminant validity results of attitude and intention scales.

Dimension	Lowest subgroup	Highest subgroup	Statistics	P
Attitude_Mean (SD)				
Total	38.99 (2.79)	54.59 (2.50)	−43.09	<0.001
Self	12.00 (1.23)	17.31 (1.11)	−35.03	<0.001
Social	16.73 (1.34)	23.40 (1.17)	−40.38	<0.001
Family	9.65 (1.18)	14.23 (0.78)	−34.32*	<0.001
Intention_Total				
Median (range)	12 (6, 13)	18 (17, 18)	−13.54^a^	<0.001

Abbreviations: SD, standard deviation.

^#^The Mann‒Whitney *U* test was used because the intention scores did not conform to a normal distribution.

a The corrected *t*-test was used because the variance between groups was not equal.

#### 3.3.2 Factorial validity

To assess the factorial validity of the instrument, we performed CFA of each multi-item scale in the UPBSC-DQ (Table 3 and Fig. S1). In this model, the ratio between χ^2^ and degrees of freedom is 3.983 (attitude) and 4.407 (intention), a value that does not exceed the limit of 5, indicating an acceptable fit between the proposed model and the observed data. All scales exhibited acceptable to satisfactory factorial validity (i.e., for attitude, CFI = 0.913, NFI = 0.899, IFI = 0.914, GFI = 0.917, RMR = 0.041, RMSEA = 0.094; and for intention, CFI = 0.972, NFI = 0.964, IFI = 0.972, GFI = 0.971, RMR = 0.011, RMSEA = 0.101).

**Table 3:** **-** Fit indices of confirmatory factor analysis (CFA) of the attitude and intention scale towards unrelated peripheral stem cell donation.

Model Fit Summary	Attitude	Intention
χ^2^	183.21	4.407
d.f.	46	8
χ^2^/d.f.	3.983	4.407
Comparative Fit Index (CFI)	0.913	0.972
Bentler-Bonett Normed Fit Index (NFI)	0.889	0.964
Incremental Fit Index (IFI)	0.914	0.972
Tucker‒Lewis Index (TLI)	0.875	0.939
Root Mean Square Residual (RMR)	0.041	0.011
Goodness-of-Fit Index (GFI)	0.917	0.971
Root MeanSquare Error of Approximation (RMSEA)	0.094	0.101
Akaike information criterion (AIC)	247.21	58.847
Browne-Cudeck criterion (BCC)	249.79	59.445
Bayesian information criterion (BIC)	369.36	112.287
Consistent Akaike Information Criterion (CAIC)	401.36	126.287

Notes: The criteria are as follows: CFI ≥0.9, NFI ≥0.9, IFI ≥0.9, TLI ≥0.9, RMR <0.09, GFI ≥0.9, RMSEA <0.100 is considered fit acceptable, CFI ≥0.95, RMR <0.05, GFI ≥0.9, RMSEA <0.08 and closer to 0.05 is considered a satisfactory fit.

### 3.4 Content validity

**Fig. 2:**
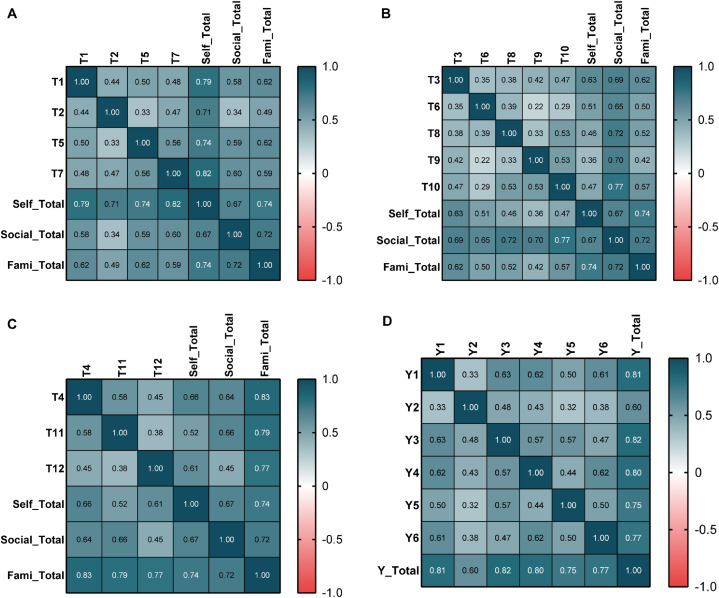
Correlation coefficient matrix plots for (**A**) self-worth, (**B**)social support, and (**C**) family support domains of donation attitude and (**D**) donation intention. Item numbers refer to the final version of the UPBSC-DQ (Supplementary File 2).

We measured the correlation between items in each domain using the Spearman correlation coefficient between items in certain domains and other domains. The correlation coefficient matrix of each scale is shown in Fig. 2(**A-D**). For the self-worth domain (Fig. 2A), the correlation of 4 items was fair (r = 0.33–0.58), and the correlation between items and the self-worth domain was moderate to good (r = 0.71–0.82). In addition, the correlation between items and other domains was fair to moderate (r = 0.34–0.62). For the social support domain (Fig. 2B), there was a fair relationship for item-Item r = 0.22–0.53, a moderate relationship for item-intro domain r = 0.65–0.77, and a fair to moderate relationship for item-extra domains r = 0.36–0.63. For the family support domain (Fig. 2C), there was a fair relationship for item-item r = 0.38–0.58, very good for item-intro domain r = 0.77–0.83, and fair to moderate for item-extra domains r = 0.45–0.66. Notably, we found higher significance for the family support content validity result, which means that it is reasonable to surmise that family support is a mean factor that influences donation activity. Broadly speaking, the results confirmed the content validity of the scales of both attitude and intention.

## 4 Discussion

To date, there is no validated instrument to evaluate cognition, attitude and intention towards UPBSC transplantation. Therefore, we developed the Cognition, Attitude, and Intention dimensions of the UPBSC-DQ and evaluated their reliability, validity, and acceptability among the participants to address this gap. Considering the inconsistent study object group and validation of methodologies across studies together with the elaborate review of corresponding means in the current manuscript, we ensured that the scope of application of this questionnaire can be extended to people outside the study object and research on donation projects other than PBSC donation.

Cronbach's α together with the corrected item-total correlation showed medium to strong internal consistency and reliability of our questionnaire. The items and scale validity indices provided evidence of the content validity of the UPBSC-DQ. Furthermore, the *t*-test, discriminant analysis and confirmatory factor analysis demonstrated the construct validity of the UPBSC-DQ. Notably, the high response rate and low percentage of unanswered questions reflected the good acceptability of the general population of students and teachers.

As a result of this study, six negative items (T5,6,9,10,12,16 in **Supplementary File 1A**) were deleted from the UPBSC-DQ whose total Cronbach's α without the items was higher than the α with the item with CITC <0.3. In particular, the case of T5, “I would worry that the donated HSC MIGHT be used on bad people and harm society instead”, is an item designed to balance the orientation of the attitude scale of this instrument. However, Chinese Confucian culture and chivalrous thought have instilled in the social consensus that leukaemia patients are among the most “weak” and “sick” of the “old, weak, sick, and disabled”. Therefore, empathy occupies first place in most people's hearts rather than considering whether the patient will harm society after rehabilitation. Compared with other questions, this question is regarded as having a relatively low impact. Thus, it is important to address this question. On the other hand, the items of the measuring instruments should be expressed without appearing to have accurate opinions or negatives to allow the respondents to understand them well [32]. In contrast, question T8, “Successful donations may BE REPORTED by the social media and GET HONORS, which is helpful to donors and will PROMPT more successful donation,” showed excellent internal consistency and reliability, indicating that the variable of social recognition has higher significance for attitude surveys.

The availability of a reliable and valid instrument in both content and construct is fundamental and necessary for social research that aims to discover factors that influence donation with regard to cognition, attitude and intention towards unrelated haematopoietic stem cell donation. The use of a nonvalidated questionnaire is likely to induce measurement error, whether in the assessment and exposure or the outcome [33]. In particular, it is crucial to validate the design of the criteria for the classification of the repent-to donate population and interventions aimed at decreasing the rate of donation repentance towards UPBSCD.

We considered cognition and attitude to be stable variables with a low likelihood of rapid change; they are potential influencing factors of donation intentions. Among the attitude item internal consistency analyses, most of the items’ maximal percentages were >25 %, except for T2, T11 and T18, indicating that the statistics might not fit a normal distribution [34]. This may be because the distribution of the sample was concentrated at the highest end of the agreement attitude scale, where participants predominantly chose 5 (extremely agree). Thus, these items were considered reliable and were not removed.

The construct validity assessment showed medium to good fitness of the adopted model. Representation of the model selected by CFA supported the consistency analysis of the 3 domains of attitudes. The primary purpose of factor analysis is to study the extraction of common factors from variable groups [35] to accelerate comprehension and interpretation of relationships among the variables considered relative [36].

This study also has limitations that should be addressed. Our study comprised a well-characterized sample that included only young adults, especially students at the WUT, with a male‒female ratio of approximately 7:3. This may restrict the universalizability of the research findings to other populations. However, the sample was heterogeneous and covered participants who differed in socioeconomic status, areas of the city or the countryside, and even nationalities [37]. Due to the limited educational level of the source population (mostly middle school and college students), the study outcomes cannot reflect social groups with other educational levels. For the cognition scale, Items R19-R21 did not capture the influence of family on individual cognition and did not influence the final results, so we deleted these 3 items. The reason may be that individuals completed the questionnaire independently from their family members or not based on the family unit. In addition, although the English version of the UPBSC-DQ was translated, the vast majority of participants used the Chinese version, which makes it impossible to determine whether language and cultural differences had an influence on the results of the study.

## 5 Conclusions

The present study provides a feasible tool for evaluating the cognition, attitude, and intention of potential UPBSCD donors. We believe that the UPBSC-DQ is suitable for evaluating cognition, attitudes, and intentions related to PBSC donation for unrelated strangers. Additionally, this standardized instrument is convenient for approaching future participants for classification according to different intention levels and for cross-group comparisons. This knowledge contributes to our understanding of volunteer attitudes towards UPBSC donation and can be used in practice to select volunteers with high donation willingness. Future studies can focus on how to intervene in cognition, attitude, and intention factors to improve the actual success rate of donation based on the UPBSC-DQ.

## Data availability statement

Data will be made available on request. Authors are willing to share any data that are used in this work. All the data that support the findings of the study are provided in the Supporting Data file.

## Ethics statement

The study was conducted according to the guidelines of the Declaration of Helsinki, and approved by the Ethics Committee of Wenzhou Medical University (Wenzhou, China). The ethics approval number: 2019-109. Informed consent was obtained.

## Data Transparency Statement

Authors are willing to share any data that are used in this work. All the data that support the findings of the study are provided in the Supporting Data file.

## Grant support

This work was supported by Wenzhou 10.13039/501100011788Philosophy and Social Science Planning Project (grant number 20wsk260), and Wenzhou Basic Scientific Research Project (grant number R2020027).

## CRediT authorship contribution statement

**NaNi Ding:** Conceptualization, Funding acquisition, Investigation, Methodology, Project administration, Software, Supervision, Validation, Visualization, Writing – original draft, Writing – review & editing. **ZhuoNi Ye:** Conceptualization, Data curation, Formal analysis, Investigation, Methodology, Project administration, Resources, Supervision, Validation, Visualization, Writing – original draft, Writing – review & editing. **XinQian Jin:** Conceptualization, Data curation, Formal analysis, Investigation, Methodology, Project administration, Resources, Supervision, Validation, Visualization, Writing – original draft, Writing – review & editing. **GuoHua Zhang:** Conceptualization, Methodology, Project administration, Supervision, Writing – review & editing. **QiuLin Yu:** Data curation, Formal analysis, Investigation. **YuPeng Liu:** Conceptualization, Data curation, Formal analysis, Investigation, Methodology, Project administration, Resources, Software, Supervision, Validation, Visualization, Writing – original draft, Writing – review & editing.

## Declaration of competing interest

The authors declare that they have no known competing financial interests or personal relationships that could have appeared to influence the work reported in this paper.
