# Supplementary material for: Assessing the cognition, attitudes and intentions of volunteers regarding unrelated peripheral blood stem cell donation: The UPBSC-DQ instrument in Chinese
Source: Heliyon. 2023 Oct 20;9(11):e20663. doi: 10.1016/j.heliyon.2023.e20663 (PMC10598484; doi:10.1016/j.heliyon.2023.e20663)
Supplement: Multimedia component 2 [file mmc2.docx]

**Supplementary File 2.** **The Final Version of UPBSC-DQ**

**Supplementary File 2_A. The final version of unrelated haematopoietic stem cell donation questionnaire.**

| **Dimension** | **Sample Item** | **Items N** |
| --- | --- | --- |
| General information | | |
| Gender | Male | 2 |
|  | Female |  |
| Age (years) |  |  |
| Phone number |  |  |
| Major | Medical-related | 2 |
|  | Not Medical-related |  |
| Family situation | One-child family | 2 |
|  | Non-one-child family |  |
| Political landscape | Member of Communist Party of China | 4 |
|  | Member of Democratic Parties |  |
|  | Member of the Chinese Communist Youth League |  |
|  | Nonpartisan |  |
| COGNITION | | |
| R1. The highest incidence of leukaemia in the population occurs in CHILDREN. | Yes/No | 2 |
| R2. Any healthy individual between 18 AND 55 YEARS of age can donate haematopoietic stem cells (HSC). | Yes/No | 2 |
| R3. HSC can also be donated by women who are PREGNANT or TRYING TO BECOME pregnant. | Yes/No | 2 |
| R4. Donated HSC is mainly used to treat blood diseases such as LEUKEMIA. | Yes/No | 2 |
| R5. Haematopoietic stem cells transportation (HSCT) can be performed only if two individuals have IDENTICAL HLA MATCH *. (*HLA match, refers to human leukocyte antigen.The success rate of HLA matching between unrelated donor patients ranges from 1 in 10,000 to 1 in 15,000.) | Yes/No | 2 |
| R6. Two individuals with a successful bone marrow matching MUST HAVE THE SAME blood type. | Yes/No | 2 |
| R7. China Marrow Donor Program (CMDP) advocates the collection of HSC from PERIPHERAL BLOOD: that is, ONLY blood collected from the donor's arm vein is needed, NOT from bone marrow. | Yes/No | 2 |
| R8. People who intend to donate peripheral blood HSC should receive subcutaneous injection of HSC mobilizer at the collection hospital or collection center ONE MONTH BEFORE the donation. | Yes/No | 2 |
| R9. Donation of peripheral blood HSC has NO ADVERSE EFFECTS on human health. | Yes/No | 2 |
| R10. The latest method of HSC extraction only requires the extraction of 100-200ML PERIPHERAL BLOOD to extract HSC. | Yes/No | 2 |
| R11. There is NO NEED FOR SPECIAL REST after peripheral blood haematopoietic stem cell donation. | Yes/No | 2 |
| R12. In the process of peripheral blood HSC donation, the volunteers DO NOT NEED TO bear any cost. | Yes/No | 2 |
| R13. The COST of the HSC donation process is entirely BORNED BY the RECIPIENT (PATIENT). | Yes/No | 2 |
| R14. Both the registration and donation of HSC are on a voluntary basis. After registration, your voluntary nature should be reconfirmed frequently. During this period, you can TERMINATE the donation AT ANY TIME. | Yes/No | 2 |
| R15. A person can donate peripheral blood HSC MANY TIMES in their lifetime. | Yes/No | 2 |
| R16. The decision to donate CANNOT BE REVOKED prior to the donation, especially after the signing of the donation consent. | Yes/No | 2 |
| R17. CMDP guarantees ABSOLUTE CONFIDENTIALITY of the personal data of HSCT donors. | Yes/No | 2 |
| R18. HSC donated by volunteers can be USED FOR FREE by patients. | Yes/No | 2 |
| ATTITUDE | | |
| T1. It is such a HONORABLE thing to be a volunteer of CMDP, which is worth being PROUD of and can REFLECT SELF-WORTH. | 1: strongly disagree / 2/ 3/ 4/ 5: strongly agree | 5 |
| T2. Now the peripheral blood HSC donation TECHNOLOGY has been very MATURE, I do NOT have too many SAFETY CONCERNS. | 1/ 2/ 3/ 4/ 5 | 5 |
| T3. Although the success rate of matching is very low, joining CMDP can bring hope to more patients. | 1/ 2/ 3/ 4/ 5 | 5 |
| T4. If my IMMEDIATE FAMILY MEMBERS and SPOUSE can enjoy the benefit of FREE BLOOD USE after my donation, I will be MORE MOTIVATED to donate HSC. | 1/ 2/ 3/ 4/ 5 | 5 |
| T5. If it is confirmed that there will be NO side effects from peripheral blood HSC donation, I will actively donate. | 1/ 2/ 3/ 4/ 5 | 5 |
| T6. Successful donations may BE REPORTED by the social media and GET HONORS, which is helpful to donors and will PROMPT more successful donations. | 1/ 2/ 3/ 4/ 5 | 5 |
| T7. After joining CMDP, if the HLA match is successful, I will decide to donate peripheral blood HSC WITHOUT HESITATION. | 1/ 2/ 3/ 4/ 5 | 5 |
| T8. REASONABLE COMPENSATION and APPROPRIATEINCENTIVES to donors play an important role in PROMOTING donations. | 1/ 2/ 3/ 4/ 5 | 5 |
| T9. Donors may NOT ask for MONETARY REWORDS from recipients in any form. | 1/ 2/ 3/ 4/ 5 | 5 |
| T10. The financial information of the Red Cross and blood collection and supply agencies will be RELIABLE, OPEN and TRANSPARENT, which will help the cause of HSC donation. | 1/ 2/ 3/ 4/ 5 | 5 |
| T11. If the donated HSC are used to HELP MY FAMILY, I will make the decision to donate WITHOUT HESITATION. | 1/ 2/ 3/ 4/ 5 | 5 |
| T12. When I want to donate peripheral blood HSC, my family was GENERALLY SUPPORTIVE. | 1/ 2/ 3/ 4/ 5 | 5 |
| INTENTION | | |
| Y1. WOULD you like to DONATE peripheral blood haematopoietic stem cells (HSC)? | 1: I won’t. | 3 |
|  | 2: I am not sure. |  |
|  | 3 point:I am willing. |  |
| Y2. Are you WILLING to UBLICIZE the KNOWLEDGE of peripheral blood HSC to the people around you? | Same as above | 3 |
| Y3. WOULD you like to ENCOURAGE your RELATIVES and FRIENDS to DONATE peripheral blood HSC? | Same as above | 3 |
| Y4. Are you WILLING to make certain changes in your LIFESTYLE in order to donate peripheral blood HSC? | Same as above | 3 |
| Y5. If you are willing to donate peripheral blood HSC, WOULD your FAMILY MEMBERS like to support it? | Same as above | 3 |
| Y6. If your FAMILY MEMBERS has donated peripheral blood HSC, are you willing to donate? | Same as above | 3 |

**Supplementary File 2_B. Deleted items from the second version. (**Items are numbered in the order of the second edition**)**

| **Dimension** | |
| --- | --- |
| COGNITION |  |
| R19. Does YOUR FAMILY know that in China, the HSC donation only requires the collection of blood from the vein of the donor's arm, not the collection of bone marrow? | |
| R20. Does YOUR FAMILY believe that there are no adverse health effects of peripheral blood stem cells donation? | |
| R21. In general, how much do you think YOUR FAMILY MEMBERS know about the above knowledge of peripheral blood HSC donation? | |
| ATTITUDE |  |
| T5. I would worry that the donated HSC MIGHT be used on bad people and harm society instead. | |
| T6. I am AFRAID that in my current state of health, I am NOT QUALIFIED to donate peripheral blood HSC. | |
| T9. Successful donation may cause EXCESSIVE ATTENTION from the society, which will AFFECT my life. | |
| T10. If I were the ONLY CHILD in my family, I would HESITATE to donate. | |
| T12. Donating HSC may affect my WORK(STUDY) SCHEDULE, and I would HESITATE to donate. | |
| T16. During COVID-19, NEGATIVE NEWS about the Red Cross in some areas made me HESITATE to donate HSC. | |

**Supplementary File 2_C. Three domains of attitude scale and contained items.**

| **Domain** | **No. of Items included** |
| --- | --- |
| Self-worth | T1. It is such a HONORABLE thing to be a volunteer of CMDP, which is worth being PROUD of and can REFLECT SELF-WORTH. |
|  | T2. Now the peripheral blood HSC donation TECHNOLOGY has been very MATURE, I do NOT have too many SAFETY CONCERNS. |
|  | T5. If it is confirmed that there will be NO side effects from peripheral blood HSC donation, I will actively donate. |
|  | T7. After joining CMDP, if the HLA match is successful, I will decide to donate peripheral blood HSC WITHOUT HESITATION. |
| Social support | T3. Although the success rate of matching is very low, joining CMDP can bring hope to more patients. |
|  | T6. Successful donations may BE REPORTED by the social media and GET HONORS, which is helpful to donors and will PROMPT more successful donations. |
|  | T8. REASONABLE COMPENSATION and APPROPRIATEINCENTIVES to donors play an important role in PROMOTING donations. |
|  | T9. Donors may NOT ask for MONETARY REWORDS from recipients in any form. |
|  | T10. The financial information of the Red Cross and blood collection and supply agencies will be RELIABLE, OPEN and TRANSPARENT, which will help the cause of HSC donation. |
| Family support | T4. If my IMMEDIATE FAMILY MEMBERS and SPOUSE can enjoy the benefit of FREE BLOOD USE after my donation, I will be MORE MOTIVATED to donate HSC. |
|  | T11. If the donated HSC are used to HELP MY FAMILY, I will make the decision to donate WITHOUT HESITATION. |
|  | T12. When I want to donate peripheral blood HSC, my family was GENERALLY SUPPORTIVE. |
