# Supplementary material for: Assessing the cognition, attitudes and intentions of volunteers regarding unrelated peripheral blood stem cell donation: The UPBSC-DQ instrument in Chinese
Source: Heliyon. 2023 Oct 20;9(11):e20663. doi: 10.1016/j.heliyon.2023.e20663 (PMC10598484; doi:10.1016/j.heliyon.2023.e20663)
Supplement: Multimedia component 3 [file mmc3.pdf]

2019-109

温州医科大学伦理委员会审查表

项目名称：非亲缘造血干细胞捐献认知、态度、意愿调查研究	
项目负责人（签字）：刘宇鹏	职称：讲师
所属院系所：公共卫生与管理学院	
联系人：刘宇鹏	联系方式（手机）：13566252653
项目起止时间：　2020 年 1 月 1 日至　2020 年 12 月 30 日	
用途：　☐项目申报　☑项目实施	
主要研究内容：<br>通过现场调查方法，对在校大学生进行问卷调查，了解其对非亲缘造血干细胞捐献相关知识的认知、态度、以及意愿等方面的相关信息。	
标本来源（注明量及次数）：温州医科大学本科生（500 人左右）	
受试者年龄层：大学生	受试人数：500 人左右
收集受试者方式（包括对照组）：<br>问卷调查。	
对受试者的预期效益、有无增加受试者额外检查次数和经济负担：<br>无。	
对受试者潜在风险、预防措施及损害的补偿：<br>无风险。	
审查意见：<br>同意<br>签章：<br>温州医科大学伦理委员会<br>2019 年 12 月 24 日	

注：另需提供以下附件：1. 项目研究方案；2. 知情同意书样本；
3. 调查类项目需提供调查问卷（调研提纲）。
